# Adolescent girls’ sexual and reproductive health information needs and barriers in Cape Town

**DOI:** 10.4102/hsag.v29i0.2650

**Published:** 2024-10-10

**Authors:** Natheerah Holtman, Million Bimerew, Katlego Mthimunye

**Affiliations:** 1 School of Nursing, Faculty of Community and Health Sciences, University of the Western Cape, Cape Town, South Africa; 2 Department of Nursing, Midwifery and Health, Faculty of Health and Life Sciences, Northumbria University, Newcastle, United Kingdom

**Keywords:** adolescent girls, adolescent sexual health, barriers to reproductive health, healthcare accessibility, information seeking, reproductive health needs, sexual and reproductive health, South Africa

## Abstract

**Background:**

The sexual and reproductive health (SRH) information needs of adolescent girls in developing countries are not being met.

**Aim:**

The study explored the sexual and reproductive health information needs, information-seeking behaviour and barriers among adolescent girls in the Western Cape, South Africa.

**Setting:**

The study was conducted in the South eastern sub district of Cape Town metropole.

**Methods:**

The study was conducted at one of the high schools in a highly populated area in the Cape Town metropole. Semi-structured interviews were conducted with 17 adolescent girls aged 16–17 years, who were recruited using purposive sampling techniques. The interviews were audio recorded and transcribed verbatim. Data were analysed using an inductive thematic analysis framework.

**Results:**

Three themes emerged from the data: (1) adolescent girls exhibit limited awareness and comprehension of sexual and reproductive health; (2) the information-seeking behaviours of adolescent girls regarding sexual and reproductive health and (3) barriers to seeking information.

**Conclusion:**

The establishment of primary healthcare facilities that are friendly to adolescents, combined with impactful reproductive health education and improved parent–adolescent girl relations, is crucial for promoting sexual and reproductive health among adolescent girls.

**Contribution:**

The study offered valuable insights into the SRH information needs, information-seeking behaviour and barriers faced by adolescent girls in accessing SRH information sources and services. These barriers include lack of knowledge about SRH and contraception, challenges within family dynamics, difficulties accessing friendly healthcare services and encountering negative attitudes from healthcare professionals.

## Introduction

Adolescent sexual and reproductive health (SRH) pertains to the mental and physical state of youth and encompasses their capacity to abstain from unintended pregnancies, unsafe abortions, sexually transmitted infections (STIs), including HIV/AIDS, and all types of sexual assault and coercion (Abdurahman et al. 2022). A survey of 70 developing countries indicated that adolescent girls had unmet needs related to SRH services, which resulted in unintended pregnancy and other socio-economic consequences (Woog et al. 2015). Approximately 16 million adolescent girls younger than 19 years give birth each year, accounting for about 11% of all births, with 2 million of these girls being under the age of 15 years (Bałanda-Bałdyga et al. 2020). Although these pregnancies occur across all brackets of socio-economic status and countries (Bałanda-Bałdyga et al. 2020), approximately 95% of adolescent pregnancies were from developing countries, especially in Africa (Odimegwu & Ugwul 2022). Sub-Saharan Africa had the highest prevalence of fertility among young women, and more than 82% of these pregnancies were unintended (Mjwara & Maharaj 2018).

In South Africa, though adolescent girls’ pregnancy rate has decreased from 30% in 1984 to 23% in 2008 (Amoo, Odimegwu & De Wet 2018), adolescent girls’ pregnancies still contributed to 13.6% of births registered in the country in 2016, a rate far higher than in any other high-income country (Amoo et al. 2018). More than 30% of adolescent girls in South Africa became pregnant every year, while 65% to 71% of these pregnancies were unplanned (Amoo et al. 2018). The fertility rate among young women remains high, with nearly 23% of them having a child during their adolescent years (Amoo et al. 2018). In 2017, the Western Cape Provincial Education Department reported that of the 79% of adolescent girls who were sexually active, only 35% were using contraceptives (Schafer 2018). In the same year, the Department of Basic Education reported that approximately 68 000 school-going learners had given birth to at least one child (Amoo et al. 2018). Although adolescent girls have the right to SRH information, they have a significant lack of such information (Jonas et al. 2018; Qolesa 2017).

There are many barriers that discourage adolescent girls from seeking SRH information from clinics, parents, friends or the community because of the perceived negative stigma in the community and the disrespectful behaviour of nurses (Odimegwu & Ugwul 2022). Literature indicates that there is a lack of parent–child communication about SRH information (Erasmus, Knight & Dutton 2020; Odimegwu & Ugwul 2022). Intergenerational discussion of sex is taboo, and many parents enforce religious beliefs to try and prevent sexual behaviour among their adolescent girls; this leaves them only able to receive information from their friends (Spadafino 2017). Most young women are not informed about the protective measures that could be taken to prevent unplanned pregnancy (Mjwara & Maharaj 2018). In South Africa, half of the adolescent girls became pregnant because of a lack of knowledge about SRH, and 55% did not understand the risk of becoming pregnant (Mjwara & Maharaj 2018). Contraceptives were misunderstood, and the few young girls who used contraceptives did so incorrectly and inconsistently (Schafer 2018). The main factor contributing to adolescent girls’ pregnancy was the lack of SRH information (Jonas et al. 2018).

The literature has shown that there is poor information-seeking behaviour of adolescent girls to acquire SRH information and understanding of how their body functions. This leads to poor decision-making skills, misconceptions about contraception and incorrect use of contraceptives, exposing adolescent girls to unintended pregnancies and complications (Indongo 2020; Jonas et al. 2019; Mpondo et al. 2018). Adolescent girls have limited access to SRH information to protect themselves against unintended pregnancy, unsafe abortion and sexually transmitted diseases (World Health Organization 2018). The health and socio-economic consequences of adolescent girls’ pregnancy are serious and wide ranging, with pregnancy and childbirth complications being the second leading cause of death among adolescent girls globally (World Health Organization 2018). The provision of various programmes aimed at addressing adolescent girls’ pregnancy, such as school-based sex education, peer education and adolescent-friendly clinic initiatives, did not produce the desired outcomes, which is attributed to the lack of relevant information being available to adolescent girls (Indongo 2020). Most studies in South Africa focussed on the SRH rights of adolescent girls and the prevalence of teenage pregnancy but no studies were found assessing the SRH information needs and barriers to information-seeking behaviour of adolescent girls in the context of this study.

This study explored the SRH information needs, information-seeking behaviour and barriers to information-seeking among adolescent girls in the Western Cape, South Africa.

## Research methods and design

A qualitative research approach with a constructivism paradigm was employed to conduct the study. The constructivism paradigm holds that knowledge is constructed through reflecting on personal experiences (Honebein 1996). Therefore, a constructivist paradigm was applied to underpin understanding the adolescent girls’ SRH information needs, barriers to accessing such information and information-seeking behaviour. A qualitative exploratory, descriptive and contextual research design was employed to explore the SRH information needs of adolescent girls at a high school in the Western Cape province. The design enabled adolescent girls who experienced early pregnancy either directly or indirectly to share their SRH information needs, information-seeking behaviour and barriers to accessing relevant information. As a health professional, the principal researcher maintained an awareness of reflexivity throughout the entire research process. She kept a book to document and reflect on her personal opinions and feelings regarding adolescent girls’ pregnancy to be aware of how these may influence data collection or data analysis.

### Study setting

The study was conducted in the south-eastern sub-district of Cape Town metropole in the Western Cape. The area is populated with low- and middle-income families, and drugs are a major problem in the community. The high school selected was a diverse school catering to all ethnic groups. The Western Cape has a birth rate of 56 per 1000 (the number of life births occurring during the year per 1000 population estimated midyear) deliveries for adolescent girls under 18 years of age, which is relatively higher than the national average (Erasmus et al. 2020). The school served a total population of 61 757 out of the total population of 310 485 (Frith 2011). The race profile of the total population is as follows: Black 6.10%, Coloured 92.63%, Indian 0.40%, White 0.10% and other 0.76% (Frith 2011). The language spoken in the area is predominantly Afrikaans (68.36%), followed by English (26.20%) and isiXhosa (2.99%) (Frith 2011).

### Study participants and sampling strategy

The population of the study were adolescent girls aged 16–17 years attending class in Grade 11, who had directly or indirectly experienced adolescent pregnancy. A total of 112 adolescent girls were in Grade 11 at the selected school at the time of the study. A sample represents a part of a whole or a subset of a larger set (Busetto, Wick & Gumbinger 2020). Purposive sampling was employed to select a diverse range of participants who could offer different perspectives and experiences and who were comfortable providing the information needed for the study (Nikolpoulou 2022). Eligibility criteria were that the participants must be registered for Grade 11 and be between 16 and 17 years of age. The class teachers made an announcement about the study in the class, and those who were interested were invited to participate in it. The interested participants were informed about the venue and the time they could meet the researcher keeping the COVID protocol. The purposive recruitment process was conducted among those students who came forward to inquire about the study, and further explanation was given about the purpose of the study and confidentiality issues. After the researcher explained the purpose of the study and all their questions were answered, the information sheet and consent form were given to them to take it to their parents to sign if they agreed for their daughter to participate in the study. Appointment was given to each participant for the interviews. Those who received informed consent from their parent could come to the appointment date for the interview, and others who could not get consent form from their parent sent a message to the researcher that they were not participating in the study. The assent form was obtained before the interview with those participants who brought the consent form from their parents and agreed to participate. Seventeen (*n* = 17) adolescent girls participated in the study.

### Data collection

Semi-structured interview guide questions were developed based on a literature review to address the objectives of the study and collect qualitative data. A pilot test was conducted on two teenage girls from the study population, who were not included in the actual study. Assent forms were handed out to the willing participants as well as a consent form that the participants gave to their parents or guardians; they returned this signed, to confirm their participation. The pre-testing of the study was used to test the appropriateness of the interview guide questions and to provide the researcher with some early suggestions on the viability of the research. It also facilitated the researcher’s building rapport with participants. The questions were open ended and probing, such as ‘Please explain your understanding of SRH?’, ‘Explain what are your SRH information needs’, ‘How do you explore SRH information?’ and ‘What challenges you have experienced when searching for SRH information?’ The interviews were conducted by a female nurse researcher at the school after class time, which gave the participants more comfort and trust to freely discuss the issue under investigation. The interviewer was a professional nurse working at an academic institution, who had no relation or connections with the study setting or participants. The semi-structured interview guide questions were posed to the participants one by one, followed by probing questions for more in-depth information. The duration of the interviews ranged between 45 min and 60 min. The concerned researcher used an audio recorder with the permission of the participants, and field notes were taken while the participant’s facial expressions and body language were observed. The interviews were continued until data saturation was achieved. At 15 interviews, the researcher noted that no further new information was emerging, but two more interviews were carried out to ensure data saturation. The interviews were conducted in English as this is the academic language of instruction, and all participants could comfortably speak it.

### Data analysis

Data were analysed inductively using the six steps of thematic analysis by Braun and Clarke (2022). The data were transcribed verbatim (manually), and field notes were read while listening to the recordings to ensure consistency of the data. Data transcription and analysis were conducted after each interview, which helped to identify any missing information and prepare for the next interview. The transcribed data were carefully reviewed to understand the core content and meaning. Codes were generated manually as per the second step of the analysis. Reflexive journaling was used to contribute to the establishment of an audit trail as evidence to support the trustworthiness of the study. Themes were developed by grouping similar codes from the data and by creating categories. The themes that emerged were compared, discussed and defined accordingly. The report was written to tell the story of the data, with direct short quotes from participants to support understanding of specific points in the interpretation.

### Trustworthiness of the study

The trustworthiness of the study was ensured by establishing credibility, dependability, confirmability and transferability. Credibility was addressed through prolonged engagement, pilot testing of the instrument, persistent observation and data triangulation. Transferability was ensured by providing a detailed description of the context, participants and research methods to show that the research process can be transferable to other similar contexts, circumstances and situations. Confirmability was addressed through documenting the audit trail, which highlighted every step of data analysis in order to justify decisions made and accurately interpret participants’ responses. The interviewing researcher used a voice recorder to ensure correct tracking of information during data transcription and kept a notebook in which all activities that were carried out during the interviews were documented. To ensure dependability the researchers reviewed and examined the research process and the data analysis to ensure that the findings were consistent and could be repeated. The findings and interpretation of data were checked and discussed with the three researchers for debriefing in order to minimise bias.

### Ethical considerations

An ethical clearance letter was obtained from the Humanity Social Science Research Ethics Committee at the University of the Western Cape. The ethical reference number is HS19/9/22. Permission to conduct the proposed research study was granted by the Department of Education for the period from 23 March 2020 to 20 September 2021. Informed consent was obtained from all participants’ parents, after which the assent form was obtained from the participants who met the research criteria. Information was provided to the participants regarding the confidentiality and anonymity of the information, and that it would be maintained during and beyond the study period. Anonymity was ensured by keeping the recordings and transcriptions of the recordings nameless. Recorded data are kept safe in a cabinet under lock and key and the soft data are protected on a desktop with a password to maintain and uphold confidentiality.

## Results

### Characteristics of the participants

The participants comprised 17 adolescent girls between the ages of 16 and 17 years. Thirteen girls belonged to the Christian religion and four to the Islamic religion. Most participants lived with their mothers or grandmothers, while one participant resided with her legal guardian and they were identified as the financial heads of the household. Most of the participants have siblings living with them, and one participant indicated that she is a teen mother.

### Themes and subthemes that emerged from the data

Three key themes and eight subthemes, with some further categories under the subthemes were generated from the data. The findings indicated that the adolescent girls’ limited understanding of SRH, their information-seeking behaviour and utilisation of reproductive health services are influenced by socio-cultural factors that also contribute to limited access to relevant information. Table 1 summarises all themes and subthemes that emerged from data analysis. Findings are illustrated with quotations stem from exact participant words from the original text. The quotes are denote with adolescent girl participant (AGP) and Grade 11 (G).

**TABLE 1:** Themes and subthemes.

Themes		Subthemes
1.	Adolescent girls’ knowledge of SRH and contraceptive awareness	1.1. Sexual and reproductive health knowledge of adolescent girls 1.2. Knowledge regarding contraceptives and their associated side effects
2.	Information channels and family influences on adolescent girls’ SRH awareness	2.1. Sources of SRH information for adolescent girls 2.2. Navigating family dynamics and information seeking
3.	Barriers to SRH information needs of adolescent girls	3.1 Perceived negative attitudes exhibited by healthcare workers towards adolescent girls 3.2 Clinic operating hours are not conducive for school-going adolescent girls 3.3 Socio-cultural factors limit access to reproductive health information and result in risky behaviour 3.4 Misinformation and misinterpretation about the usage of contraception

SRH, sexual and reproductive health.

#### Theme 1: Adolescent girls’ knowledge of sexual and reproductive health and contraceptive awareness

Many adolescents become sexually active while very young, and this poses a risk to them because they become vulnerable to issues such as HIV infection, unintended pregnancy and maternal complications. Early initiation of sexual activity is commonly associated with unsafe sex because of a lack of knowledge and understanding of SRH, limited awareness about contraception and lack of power to negotiate contraception.

*Subtheme 1.1: Sexual and reproductive health knowledge of adolescent girls:* The lack of knowledge about SRH is a key factor that contributes to adolescent pregnancy. Participants did not have knowledge of how to protect themselves from sexual ill health despite most being sexually active. Early sexual debut is often associated with negative psychosocial and health outcomes. In many circumstances, adolescent pregnancy is accidental, and this is often the result of risky sexual behaviour, which mostly arises because of the lack of reliable information about contraceptives. The participants revealed their lack of knowledge about SRH:

‘I do not know much about sexual reproductive health, it is like they are giving you advice not to have sex and if you have sex like what you must use to prevent pregnancy.’ (P14, AGP14, G11)

‘All I know about sexual and reproductive health is that it is about having sex, but I’m still too young to have a baby. I know a person can use condoms to prevent falling pregnant, but further than that, I don’t know much about SRH.’ (P4, AGP4, G11)

*Subtheme 1.2: Knowledge regarding contraceptives and their associated side effects:* Participants demonstrated basic knowledge of various aspects associated with contraception. They demonstrated familiarity with three different types of contraceptives, including barrier methods such as condoms as well as oral and intramuscular contraceptives, contraceptive methods, the benefits of contraception and side effects. They also demonstrated basic awareness of the associated benefits and side effects of contraception use.

*Category 1: Knowledge of contraceptives and contraceptive methods:* Despite a variety of available contraception methods, a significant knowledge gap regarding contraceptives, their various methods and their correct usage remains among adolescent girls. They were not sure whether the injection or oral contraceptives were appropriate for them:

‘No. I don’t have a clue about contraceptives. I’ve heard about female contraceptives but I haven’t seen one and I don’t know how to use it. I actually don’t know how the injection and condom work or anything about the side effects. I am in a relationship and my boyfriend uses a condom.’ (P9, AGP9, G11)

*Category 2: Benefits of contraception:* Access to and correct use of contraception offers an array of benefits, not only for adolescent girls but for society. Beyond family planning and preventing unwanted pregnancies, contraception empowers adolescent girls and provides them with the autonomy to make decisions about their futures. The participants exhibited basic knowledge of the benefits of contraception but appeared to lack confidence in their responses:

‘I just know that with the injection, if you are using it then you can’t have babies in future.’ (P11, AGP11, G11)

‘I only know that like contraceptive is there to help you to avoid having babies and stuff like that.’ (P7, AGP7, G11)

‘The people I know who use the injection couldn’t fall pregnant after using it …’ (P4, AGP4, G11)

*Category 3: Associated side effects:* The side effects of contraception and the mechanism of action to prevent pregnancy remain blurred and misunderstood. Participants were misinformed about the side effects of the contraceptives – and they were more concerned about the side effects than the benefits of using them. Participants were asked about their knowledge regarding the side effects of contraception, and many of them lacked insight:

‘I want to know about contraception and the side effects. I just think that they should make this type of information easier to access.’ (P8, AGP8, G11)

Changes in weight was the most common side-effect mentioned by the participants. Their limited knowledge of side effects increased their resistance to using contraception. Fear of the unknown is a barrier for adolescent girls in accessing contraceptive services:

‘Sometimes contraception makes you fat or thin, your facial expression changes, you are not the same person. You’re not the same with your partner as you were before, because there’s something else happening with your hormones.’ (P14, AGP14, G11)

‘Going on injection or the oral contraceptives in the future, like in long-term, it will prevent me from having babies or it can cause something to my body.’ (P17, AGP17, G11)

#### Theme 2: Information channels and family influences on adolescent girls’ sexual and reproductive health awareness

Adolescent girls have an interest in searching for information about the risks of contraception use and side effects, sexual experiences and STIs. The easiest way for them to obtain information without being judged is in their private space. Some participants reported seeking this information from various sources, including their families, friends, libraries, schools, places of worship, clinics and the Internet. Participants reported that they heard about oral contraceptives and injections from friends and family but did not have much knowledge about them:

‘I have only heard about the oral contraceptives and injection from family and friends, but I don’t know a lot about it.’ (P8, AGP8, G11)

‘I just know what my friend says. She first went on the oral contraceptives and then it is better than a needle. Oral contraceptive or the injection, that’s the only choice.’ (P6, AGP6, G11)

*Subtheme 2.1: Sources of sexual and reproductive health information for adolescent girls:* Most participants indicated that they looked for information from different sources, including the Internet, library, life orientation class, family members, friends or peers and the local clinics:

‘I will ask my mommy about any information I need. Sometimes I will go on the Internet just to search the consequences of using contraception. But basically, my mommy. I am the closest to her.’ (P1, AGP1, G11)

‘I would go on the Internet and Google whatever comes to mind about my SRH.’ (P17, AGP17, G11)

‘I’m going to Google, which is the first thing I would do. I would ask my mommy because she’s now past that stage (experienced) and so on. That’s my only options. I trust Google.’ (P11, AGP11, G11)

‘Most of the time I would go to the library which is just next to the clinic, to look for information.’ (P7, AGP7, G11)

‘… I don’t talk about my SRH with anyone, I am a *bietjie* [*little bit*] nervous. I think I would rather use the Internet, but I don’t know if the information is truthful, it must probably be.’ (P3, AGP3, G11)

‘I stay with my eldest sister. I’ll ask her most questions about situations I have already experienced. If I’ve experienced something like having sex without a condom and there’s a chance, I could fall pregnant, I would ask her what can I do and how will it affect me?.’ (P16, AGP16, G11)

*Subtheme 2.2: Navigating family dynamics and information seeking:* The adolescent girls had differences in their information-seeking behaviour. Participants expressed that they were scared to seek SRH information because of religious beliefs and had differences in their frequency of looking for SRH information:

‘I don’t live with my parents; I live with my grandparents, but I don’t talk with them [*about*] such stuff. I’m scared to talk to them.’ (P15, AGP15, G11)

‘… my family are very strict with religion; they say no sex before marriage, but I want to have sex before marriage. I can’t speak to them because they’re so judging, they judge you straight.’ (P4, AGP4, G11)

‘… I do not search for SRH information every day, I search for information sometimes when I see something is wrong with me, then I will ask my mommy or go look for information and stuff like that.’ (P17, AGP17, G11)

‘… I look for information once a month. Information I look up is like what must you use to have safe sex? How to prevent getting pregnant? I was told by my elder cousin that I must go to the clinic and get the oral contraceptives or injection or use a condom to prevent getting pregnant.’ (P15, AGP15, G11)

#### Theme 3: Barriers to sexual and reproductive health information needs of adolescent girls

Despite many adolescent girls showing interest in knowing about their SRH, they did not seek information because they felt embarrassed and feared being judged. Discussing sexual-related matters is considered a taboo, and it is not entertained in the family and society. Participants mentioned that they were too shy and embarrassed to ask questions, and others were not interested in seeking SRH information. Because of the lack of information-seeking behaviour, adolescent girls are not making informed decisions or feel limited in their rights to make decisions. In most circumstances, adolescent girls are not given the information they need about which contraception to use.

*Subtheme 3.1: Perceived negative attitudes exhibited by healthcare workers towards adolescent girls:* Adolescent girls feel discouraged from visiting the clinic because of the discrimination against them because of their age. Healthcare professionals exhibit unfriendly behaviour, and the nurses’ negative attitudes make the healthcare system unconducive for adolescent girls, who then rather seek information from their peers, which may sometimes be incorrect:

‘… [*W*]hen you go to the helpdesk, you just want to know if you can get an appointment. It is your first time; you don’t know what to do. They’re not going to guide you, do this before you can do that. No, they’re going to be sarcastic and if you say something and then you are regarded as rude, and they want to put you out.’ (P10, AGP10, G11)

‘When I went to the clinic for the 3-month injection, they had the oral contraceptives and the two-month injection. I told the nurse I want the two-month injection, but she told me the oral contraceptives are better because it teaches me responsibility. I told her that previously I’ve been using the oral contraceptives, but I fell pregnant. She told me I didn’t have a choice, because that’s how she feels it is going to teach me responsibility. You are forced to take whatever the nurses give you. Even if you want to know information from them, they don’t tell you.’ (P1, AGP1, G11)

‘At the clinic, the nurses don’t give you all the information you seek to know and need to choose a contraceptive method from. They give you a choice of their own.’ (P6, AGP6, G11)

The participants expressed dissatisfaction regarding how the healthcare workers addressed them. The attitudes and behaviour of the healthcare workers towards adolescent girls were unfriendly, hostile, rude and judgemental. Adolescent girls stayed away from the clinics, fearing nurses’ judgemental attitudes and being reprimanded by them. The implications of the nurses’ judgemental attitudes are that they can deprive adolescent girls of obtaining appropriate health information to enable them to make informed decisions:

‘… nurses at the clinic don’t help you because I’ve noticed that they ask the girls what they are doing there, and if they are pregnant then they ask questions like, ‘Why do you open your legs early?’ and stuff like that.’ (P8, AGP8, G11)

‘… [*W*]hen you go to a nurse, the nurse would go to another nurse and say, ‘Look at this child, she just got pregnant, why would she do that? She’s so young. You know these children don’t have manners’.’ (P9, AGP9, G11)

‘… at the clinic everyone is unapproachable. You want to ask something, but the way their faces look, it is like they are thinking ‘I don’t have time, just leave me alone’. They are moody sometimes.’ (P10, AGP10, G11)

*Subtheme 3.2: Clinic operating hours are not conducive for school-going adolescent girls:* The local clinics are usually in close proximity to most adolescent girls; however, the appointment system at the clinic was unfavourable for learners. The adolescent girls were given appointments to access their SRH services early in the morning or late in the afternoon, but this time was not conducive as school starts early and ends late. The clinics are not open for SRH services on the weekend:

‘… at the clinics, they said they have time, maybe at 7 o’clock till 8 o’clock in the morning, where they only take five people to make an appointment for the contraceptive injection. If you come after that time, you can’t make an appointment. That’s also a problem because like two years ago they would always do family planning on a Friday at 12 o’clock, but we come out of school half past 12 pm. And now it is on Wednesday and Thursday from 7 am to 8 am, but that’s the time when we must go to school.’ (P7, AGP7, G11)

Although the adolescent girls were told to come in the afternoon, they did not get help at the clinic in the afternoons after school; most of the time they were turned away and told to come back the next day:

‘… when I went to the clinic after school, they turned me away and they said I must come back the next day: ‘You must come early in the morning’. So I told them I was at school and couldn’t come earlier, but they still turned me away and they didn’t help me.’ (P13, AGP13, G11)

‘… if I come out of school then I get home maybe like half past 3 pm, then I go to the clinic, they tell you ‘No, no you kids are always in a hurry and you can never do your stuff in time’. Then they judge you, scold you, and tell you no, you must come the next day, because they have their own time and want to go home early.’ (P9, AGP9, G11)

Adolescent girls defaulted on their contraception because the appointment times were not convenient for them to attend the clinic without missing school:

‘… I stop my contraceptive, because I come out of school half past 3 pm, but the clinic closes at 4 o’clock, and I won’t be able to get to the clinic in time.’ (P8, AGP8, G11)

The appointment-based system at their local clinics is not suitable for learners, as it is affecting their school attendance, and the long waiting time at the clinics contributed to the low utilisation of SRH services. The nursing staff did not show empathy or consideration towards the adolescent girls.

*Subtheme 3.3: Socio-cultural factors limit access to reproductive health information and result in risky behaviour:* Adolescent girls fear seeking SRH as they are concerned about community recognition, judgement and potential parent involvement. Some participants felt uncomfortable accessing SRH information or services for religious reasons, resulting in their lack of interest in seeking the SRH information and services:

‘I am afraid to talk about it because every time you talk to the family, they make their eyes [*eye rolling*] as if to say I know what you do.’ (P4, AGP4, G11)

‘… I can’t ask anybody at home. Like you can’t go to your priest and ask him about this and that. It is very wrong; you can’t do it.’ (P7, AGP7, G11)

Most of the participants expressed that they do not have open communication about SRH topics with their parents or grandparents, because such topics are regarded as offensive and disrespectful and have consequences. Furthermore, parents do not create a platform for their adolescents to talk freely about sex or any other topic about SRH. Traditions are inherited from generation to generation, and talking about sex is considered to promote promiscuity, so the cycle continues:

‘… in the house, my mommy does not want to hear about that type of stuff. She usually accuses me of such stuff. I said to her that if you accuse me of something, then I’m going to do it. There’s no communication between us, we don’t talk about such things, nothing like that.’ (P12, AGP12, G11)

‘… If I talk about sex, they would throw me out of home. They’ll disown me from the family. They’re very strict.’ (P10, AGP10, G11)

‘… my parent will kill me if I raised such issues to discuss. It is just a tradition; it says you can’t talk about sex. Like my parents, they wouldn’t sit down with me and talk about the consequences of having sex. They would just say okay no boyfriend, nothing. So that comes with that saying no sex, nothing.’ (P3, AGP3, G11)

There is a stigma attached to the use of contraception by adolescent girls and surrounding adolescent pregnancy. The stigma is influenced by social, religious and cultural norms. Adolescent girls were more concerned about how people would react towards them should it become known that they were using contraception. Contraceptive use is associated with sexual intercourse, which is forbidden before marriage in many cultures and religions:

‘… yes, they will always say ‘Why does “X” go on contraception, isn’t she Muslim? Why didn’t she wait for marriage before having sex?’. For me, she is just a person like any other person who has sexual feelings. You can’t like kill yourself just because you want to have sex and you want to try something new, because your religious community judges you or your family’s reputation is degraded. So, to uphold your family’s reputation you are not going to do such thing, but you’re going to suffer at the end of the day.’ (P10, AGP10, G11)

Most adolescent girls were reluctant to visit clinics because they felt that nurses violated their privacy and confidentiality by threatening to report them to their parents, and they were also afraid of being recognised at the local clinic by neighbours, who posed a threat of reporting them to their parents:

‘… somebody [*from the community*] might see me there and tell my mommy. So that prevents me from going to the clinic.’ (P3, AGP3, G11)

‘When I went for this contraception and the neighbours [*the community*] may see you, and gossip about you, this one about that one and the whole road knows [*aware of private information*] and then they say you are pregnant, but you’re not.’ (P10, AGP10, G11)

*Subtheme 3.4: Misinformation and misinterpretation about the usage of contraception:* Misinformation and misinterpretation about the use of contraception affect the way the information is perceived and shared among adolescent girls. The negative perception about contraceptives was influenced by misinformation received from various sources, highlighting the influence that others can have on decision-making around contraceptive use:

‘By using contraceptives you preventing a blessing, because a baby is a blessing. You can’t go on contraception before the time, unless you are older. Don’t prevent a blessing, because a baby is a big blessing. No matter your age, the time – you will never know what’s going to happen, but don’t go on family planning. I don’t believe in that.’ (P14, AGP14, G11)

Certain misconceptions and myths exist that influence adolescent girls’ decisions around their use of contraception. Many females believe that Depo-Provera [*injection*] causes infertility and that oral contraception is risky because females fall pregnant easily while using it.

Some participants see contraception as a means of cleansing the female body and getting rid of ‘dirty blood’, which may have been caused by her experiencing amenorrhoea. Other participants believe that the use of injectable contraceptives causes infertility and that they would not be able to have a baby in future:

‘My mommy believes that the menstruation blood stays behind in the body when the injection is used and calls it ‘dirty blood’. The dirty blood needs to be removed.’ (P1, AGP1, G11)

‘I just know like with the injection, if you are using it, then you can’t have babies in future. If you want to get pregnant in the future, you can’t get pregnant because of the contraceptive injection.’ (P11, AGP11, G11)

The misinterpretations of SRH information are being incorrectly conveyed from generation to generation in lower socio-economic societies, succeeding in perpetuating a dangerous cycle. It is a common misinterpretation that a female cannot conceive after using contraception, because of damage to the reproductive health system caused by the contraceptive’s side effects.

## Discussion of findings

This study highlighted that adolescent girls face several challenges. It is important to understand that adolescent girls are at greater risk of sexual abuse, unwanted pregnancies, STIs, social stigma, and alcohol and drug abuse. Adolescent girls did not have adequate knowledge about SRH, particularly about contraception. The sources of information about SRH from family or friends are usually incorrect, or they did not always understand the information received on how to protect themselves from falling pregnant and complications. The lack of appropriate guidance created a sense of anxiety and vulnerability among adolescent girls. In line with our findings, Brody (2018) identified that high school learners require the most recent information about contraception, as well as easy access to SRH services. Similarly, Qolesa (2017) reported that the lack of understanding of SRH is considered one of the major contributing factors in adolescent girls’ pregnancy.

The study findings indicated that although adolescent girls know about certain types of contraception, they were uncertain about the contraceptives’ mechanism of action within the female body, which includes the side effects of contraception and the menstrual cycle. The exposure to unpleasant side effects and uncertainties about side effects played a key role in disapproval of the effectiveness of contraception. Some of the side effects of contraceptives reported were weight gain and infertility. However, Villines (2020) reported that numerous studies have shown that contraception does not cause weight gain or infertility.

The study identified a lack of reliable and accurate information about SRH, notably about pregnancy prevention, and the use of condoms during intercourse. Given the high burden of adolescent girls’ pregnancies and diseases among young adolescent girls, there was less emphasis on SRH education to reduce risky behaviour. The previous study found that most adolescent girls would like more information about family planning methods (Jonas et al. 2018). Adolescent girls expressed that their information-seeking behaviour was affected by many societal factors. They were nervous and feared going to their local clinic in search of SRH information and services because the nurses were judgemental and often they felt bullied and unsupported by nurses at the clinic. This finding is congruent with those of Narker (2022), who stated that nurses were unsupportive towards adolescent girls’ information-seeking behaviour in decision-making about their SRH.

Adolescent girls were led to believe that even just enquiring about information about sex was considered bad, sinful and inappropriate for their age. This discouraged the adolescent girls from seeking information about SRH, which contributed to their poor information-seeking behaviour. Kitching, Yates and Koch (2019) reported that adults scare and shock adolescent girls by communicating with them and conveying how bad sex is. Parent–adolescent girls’ communication about SRH and sex has a significant influence on adolescent girls’ behaviour in making responsible decisions regarding their SRH and attending clinics for those services. According to Duby et al. (2022), parent–adolescent girls’ communication is particularly important in reducing the rate of adolescent girls’ pregnancy.

Adolescent girls’ curiosity about sex stems from conversations and interactions with their friends. They felt comfortable sharing information with their friends who had experience. The findings indicated that most participants had close friends whom they could trust to share their personal information with. According to Qolesa (2017), adolescents spend more time with friends and peer groups than with their parents, which can affect their choices and decisions. In some cases, it was noted that adolescent girls only seek SRH information after the problem becomes severe and they cannot hide it anymore.

The perceived unprofessional attitudes and behaviour of nurses contributed to the unfriendly environment at the local clinic. Adolescent girls feel embarrassed and stigmatised when attending clinics for contraception. The participants reported that nurses are rude, humiliate them in front of others and are judgemental, resulting in adolescent girls’ reluctance to access clinics for SRH information and services. Nurses threatened the adolescent girls that they would report them to their parents. These findings are consistent with those of a study conducted by Nmadu (2017), who identified that the judgemental and unfriendly attitudes of healthcare workers towards adolescent girls seeking information related to SRH also violated the rights of these adolescent girls. Jonas et al. (2020) provided evidence that nurses shout at adolescent girls and humiliate them by saying they are too young for contraception in front of everyone at the clinic.

The study identified that adolescent girls were expected to be at the clinic for contraceptives in the morning during school time. However, there would be no time for travel between the clinic and school, and the entire experience would be rushed, with no time to exercise their information seeking for a clearer understanding and decision-making regarding their SRH. Adolescent girls expressed that nurses turn them away if they do not come at the prescribed date and time. They were only allowed to visit clinics on certain days per week, in the morning between 7 am and 8 am – at the time they were supposed to be at school. The clinic’s operational hours usually correspond with the time they are meant to be at school (Nmadu 2017). The long waiting times were another factor contributing to the low utilisation of SRH services. Adolescent girls visiting the clinic after school felt that nurses were not being considerate towards them: either they were turned away, or they were made to join the long queues even though their visit to the clinic was after a long day at school. The non-flexible operating hours of the clinic were not favourable and hindered adolescent girls’ use of SRH services. Mokomane et al. (2017) reported that some of the health facilities’ opening and closing hours are not convenient for school-going adolescents.

Our study further highlighted that socio-cultural factors, which include societal stigma and parent–child communication barriers, affect access to SRH information. Traditional, cultural or religious beliefs affect many things in an adolescent girl’s life, including access to SRH information and services. Talking about sex or anything related to sexuality was seen as taboo because of their culture and/or religion. Many cultures and religions are strict about talking about sexuality or having sex before marriage; these restrictions cause significant barriers that inhibit adolescent girls from exploring their sexuality and make them vulnerable.

Although adolescent girls perceived parents as the primary source of SRH information, parents are usually not willing to discuss sex with their children. Traditionally, if young girls enquired about SRH then they were believed to be promiscuous. Loi et al. (2019) identified that contraceptive use was associated with a promiscuous lifestyle. This study identified that many adolescent girls did not know that it was considered acceptable by many people outside of their religion and/or culture to search for information about SRH. The community disapproved of adolescent girls accessing SRH services; as a result, the girls were scared of being recognised by members of the community, who may know their families, if they attempted to seek SRH services at the clinics. A similar study by Nmadu (2017) identified that adolescent girls were afraid of being seen by family and neighbours when utilising SRH services.

Adolescent girls prefer to seek information from their friends rather than their family members because they are afraid of being questioned as to why they are interested in searching for SRH information. It is believed that it would be disrespectful to discuss SRH matters because it is seen as taboo – thus adolescent girls have many unanswered questions concerning SRH. Qolesa (2017) reported that parents feared promiscuity, adolescent girls’ pregnancy and all the challenges associated with it, so when adolescent girls spoke to their mothers, they did so with caution. A similar study indicated that older persons regard it as culturally immoral and disrespectful to talk about sex with their grandchildren (Narker 2022).

This study revealed that adolescent girls were influenced by religious norms, which impacted their accessibility to SRH services. These greatly restricted adolescent girls who belonged to staunchly religious or cultural families. In many religions and cultures, it is believed that sexual relations should only occur within marriage; otherwise it is considered sinful. Unmarried adolescent girls are particularly affected by the stigma around contraceptive use because of religious beliefs about premarital sex and social norms. Loi et al. (2019) reported that religious beliefs and socio-cultural norms continue to contribute to the condemnation of premarital pregnancy and contraceptive use. Religious and cultural taboos prevented parents from discussing SRH matters with their adolescent girls, which meant that limited guidance and support were available to the girls.

The findings echoed the understanding of Fakudze (2018) that the reasons for adolescent girls not using contraception include their fear of being stigmatised. The study reveals that poor SRH consultations and lack of SRH knowledge led adolescent girls to misinterpret the information about contraceptive use. Yakubu and Salisu (2018) reported that during consultations, the clinician does not explore the fear that adolescent girls have regarding contraception, causing inadequate SRH education and misconceptions. Misinterpretation of the information about oral contraceptives was a big barrier for adolescent girls, as it deterred their use of contraception. Some adolescent girls held the misconception that oral contraceptives were effective as soon as they started using them, which is not true. A female’s natural hormones in the body need to become familiar with the artificial hormones of the oral contraceptive before it becomes effective. It takes at least 1 week for the hormones in oral contraceptives to work with a woman’s natural hormones to prevent ovulation (Brody 2018). The study revealed that information about oral and injectable contraceptives was misinterpreted. Participants perceived that oral contraceptives could cleanse the body from ‘dirty blood’ stored in the body during amenorrhoea. Polis, Hussain and Berry (2018) indicate that some women perceived amenorrhoea to cause a build-up of ‘dirty’ or ‘blocked’ blood, which in turn was perceived as causing blood clots, fibroids, emotional disturbances, weight gain, infertility or death. Some participants believed from what they heard that the Depo-Provera injection could cause infertility, but they had no evidence to support this. This finding is in line with Mwaisaka et al. (2020), who report that adolescent girls perceived the use of contraceptives as jeopardising future fertility.

In addition, adolescent girls are faced with many socio-economic challenges, including the lack of support and guidance from their parents or caregivers, which render them vulnerable to many risky behaviours, such as engaging in commercial sex for monetary gain to support their family or their lifestyle habits. Similarly, the findings of Ruiters (2022) identified that young girls were lured to sex parties and given money or expensive clothing in exchange for sex with men. In such risky engagements, these girls often do not use condoms or contraception to protect themselves from STIs or pregnancy – and this would often be at the demand of the paying customer. Kitching et al. (2019) suggested that adolescent girls with no parental guidance are more at risk of exploitation by sexual predators such as ‘sugar daddies’. Ruiters (2022) reported that young girls are unwittingly becoming trapped in the sex trade when offered money, drugs or alcohol. Those adolescent girls who are addicted to drugs and/or alcohol have no clear way of thinking.

The context of this study is also known for its illicit drug use, where young girls are influenced to engage in drug and alcohol use, which contributes to unsafe sexual activities.

### Limitations of the study

Recruiting participants was found to be challenging because of the coronavirus disease 2019 (COVID-19) national lockdown. This prevented the principal researcher from speaking to the population group as a whole when presenting the research topic and what the study entailed. Adolescent girls’ pregnancy is a very sensitive topic to discuss; as a result, some sensitive information may have been withheld from the researcher, mainly because of religious reasons. Also, participants could have shared information which they thought the researcher wanted to hear, which might not necessarily have been the truth. Therefore, the findings of the study may not be transferable to all other contexts.

### Recommendations

To meet the adolescent girls’ SRH information needs and improve information-seeking behaviour, the availability and accessibility of SRH information resources at the school level must be ensured. There is also a need to address the negative attitudes of healthcare professionals and establish friendly clinics for school-going girls. Parent–adolescent girls’ communication, in general, needs to be improved, and quantitative research at a larger scale will be useful to include the wider community and design a comprehensive policy plan to improve adolescent girls’ SRH services.

## Conclusion

Our findings show that adolescent girls have a low level of knowledge and awareness regarding SRH. Adolescent girls face barriers in terms of health services and socio-cultural and family dynamics, regarding access to SRH information sources and services. Understanding and addressing the barriers are essential to enable adolescent girls to exercise their rights to SRH information and services. There is, therefore, a need to focus on adolescent girls’ needs relating to SRH and to establish youth-friendly healthcare services that address their needs.

There is a need for engaging with nurses on the rights of adolescent girls to SRH services, including contraceptive information and counselling services, as well as for improving parent–adolescent girls’ communication. The school also needs to relook at the curriculum in terms of the appropriateness and adequacy of the content of the SRH topics.
